# Understanding and modeling nerve–cancer interactions

**DOI:** 10.1242/dmm.049729

**Published:** 2023-01-09

**Authors:** Thanh T. Le, Madeleine J. Oudin

**Affiliations:** Department of Biomedical Engineering, 200 College Avenue, Tufts University, Medford, MA 02155, USA

**Keywords:** Cancer, Innervation, Models

## Abstract

The peripheral nervous system plays an important role in cancer progression. Studies in multiple cancer types have shown that higher intratumoral nerve density is associated with poor outcomes. Peripheral nerves have been shown to directly regulate tumor cell properties, such as growth and metastasis, as well as affect the local environment by modulating angiogenesis and the immune system. In this Review, we discuss the identity of nerves in organs in the periphery where solid tumors grow, the known mechanisms by which nerve density increases in tumors, and the effects these nerves have on cancer progression. We also discuss the strengths and weaknesses of current *in vitro* and *in vivo* models used to study nerve–cancer interactions. Increased understanding of the mechanisms by which nerves impact tumor progression and the development of new approaches to study nerve–cancer interactions will facilitate the discovery of novel treatment strategies to treat cancer by targeting nerves.

## Teaser

**Summary:** This Review summarizes what is known about the types of nerves in solid tumors, the experimental models used to study nerve–cancer interactions and the translational implications of these interactions.

## Introduction

The tumor microenvironment (TME) has a well-established and critical role in driving cancer progression. The TME is rich in a range of cell types, such as resident epithelial and stromal cells, immune cells, the vasculature and lymphatics, as well as the extracellular matrix, which provides structure and support to tissues. Peripheral innervation was first detected in the TME nearly 40 years ago (Batsakis, 1985). Since then, increased nerve density has been reported in histological studies of multiple solid cancer types: pancreatic (Hirai et al., 2002), prostate (Ayala et al., 2008), ovarian (Allen et al., 2018), gastric (Zhao et al., 2014), colorectal (Albo et al., 2011), head and neck (Madeo et al., 2018), lung (Shao et al., 2016) and breast (Huang et al., 2014). For example, although benign breast tissues are sparsely innervated, over half of high-grade breast tumors are extensively infiltrated by nerves (Huang et al., 2014). This association of nerve density with poor outcomes has prompted further investigation into the mechanisms that drive increased innervation and the effects that nerves have on tumor progression. Nerve infiltration is thought to occur early in tumor progression, and pain can be the earliest sign of cancer in patients (Mantyh, 2006). Peripheral nerves can support tumor growth, as well as dissemination to distant organs (Magnon et al., 2013; Saloman et al., 2016; Zhao et al., 2014). As a result of these active roles, tumor innervation provides new opportunities for detecting and treating cancer (Demir et al., 2020). In this Review, we aim to summarize the current literature on the roles of peripheral innervation in cancer. We focus on the types of nerves within solid tumors and review what is known about how increased innervation occurs. We review the effects of different nerve types on cancer phenotypes *in vitro* and *in vivo*, and discuss the strengths and weakness of experimental models to study nerve–cancer crosstalk.

## Solid tumors are innervated by distinct nerve types

**Fig. 1.**
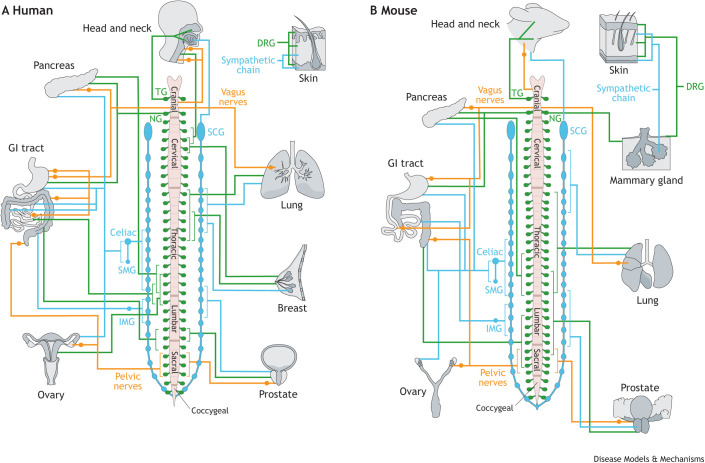
**Peripheral nerve supply to organs affected by solid tumors.** (A,B) Human (A) and mouse (B) nerve supply. The images depict sections of the peripheral nervous system, from top to bottom: cranial, cervical, thoracic, lumbar, sacral and coccygeal. Ganglia of the peripheral nervous system are organized symmetrically, and organs often receive innervation from the same ganglia from both sides of the body. Solid tumors can also be innervated by the enteric nervous system, but this is not shown in the figure as this system is regarded as separate from the peripheral nervous system. Sensory, sympathetic and parasympathetic nerve fibers are marked in green, blue and orange, respectively. DRG, dorsal root ganglion; GI, gastrointestinal; IMG, inferior mesenteric ganglion; NG, nodose ganglion; SCG, superior cervical ganglion; SMG, superior mesenteric ganglion; TG, trigeminal ganglion.

When investigating the role of nerves in cancer progression, it is important to know which nerves are present within the healthy tissue the tumor originates from. Organs in the body receive input from different types of nerves (Box 1). Furthermore, nerves from different sections of the peripheral nervous system (PNS) are highly distinct. Development of each section is regulated by different transcription factors – the cranial (CN) section is controlled by HOX1-5, cervical (C) section by HOX5-9, thoracic (T) section by HOX9-10 and sacral (S) section by HOX10-13 (Lippmann et al., 2015) – and this regional specificity is necessary for neural function (Kriks et al., 2011). Thus, the nerve supply to each organ is unique and warrants careful consideration during experimental design. We describe the identity and origins of the nerves present in organs that can develop solid tumors in human and mouse (Fig. 1).

**Box 1. Overview of the peripheral nervous system**

The peripheral nervous system (PNS) comprises all the nerves outside of the central nervous system (CNS). Developmentally, PNS and CNS nerves originate from cells in the neural plate of the ectoderm. The neural plate folds to form the neural tube, which develops into the CNS, and the neural crest, which develops into the PNS. PNS progenitor cells migrate throughout the body to innervate organs. PNS neural cell bodies are primarily organized in clusters called ganglia that are distributed in specific sites in the body. The spinal cord connects the PNS and CNS with pairs of spinal nerves sprouting from five different segments: eight cervical (C1-C8), 12 thoracic (T1-T12), five lumbar (L1-L5), five sacral (S1-S5) and one coccygeal. There are also 12 cranial nerves (CN I-XII) emerging from the brain or brainstem that provide innervation to the head and other organs. Together, these spinal and cranial nerves form the PNS.

The distribution of the PNS around the spinal cord is visualized in Fig. 1. The PNS is divided into two systems, somatic and autonomous, encompassing three types of peripheral nerves: motor, sensory and autonomic. The somatic nervous system oversees voluntary control of the body and is made up of motor and sensory neurons. Motor neurons reside in the spinal cord and innervate skeletal muscles and glands. Sensory neurons mainly reside in the nodose ganglia (NG) in the cranial region and in dorsal root ganglia (DRG) that are closely attached but outside of the spinal cord and that innervate organs to carry sensory information to the brain. There are 31 pairs of dorsal roots in the human body, one for each spinal nerve. The autonomous nervous system is composed of sympathetic, parasympathetic and enteric neurons that control involuntary bodily functions. The sympathetic nervous system stimulates the fight-or-flight response, and sympathetic ganglia form two long chains that run parallel to either side of the spinal cord until they converge at the coccygeal nerve. The parasympathetic nervous system stimulates the rest-or-digest response. Parasympathetic nerves originate mostly from cranial nerves (vagus nerves) and three spinal nerves S2-S4 (pelvic nerves). There is no central location for these ganglia, which instead form near or inside their target organs (intramural ganglia). The enteric nervous system, which controls innervation of the gastrointestinal tract, contains both autonomic and somatic nerves. However, it is often viewed as a separate system due to its complexity and ability to act independently of both systems.

The gastrointestinal (GI) tract receives innervation from the enteric nervous system and all three main peripheral nerve types (Box 1). In the abdomen, nerves from the thoracic and lumbar (L) segments of the sympathetic chain join to form three ganglia – celiac, superior mesenteric and inferior mesenteric – from which nerves innervate the GI tract. Parasympathetic innervation originates from both CN X (also called the vagus) and the pelvic nerves (Uesaka et al., 2016). Sensory innervation of GI track arises via the cranial and spinal nerves with neurons in the nodose ganglia (NG) and dorsal root ganglia (DRG) T10-L1 and L4-S1, respectively (Robinson et al., 2004; Spencer and Hu, 2020; Tan et al., 2008).

The ovaries, prostate, pancreas and lungs are also innervated by all three peripheral nerve types, with autonomic innervation being dominant. The ovaries receive parasympathetic innervation from the pelvic nerves, sympathetic nerves from the celiac and superior mesenteric ganglia, and sensory nerves from DRG T10-L1 (Burden et al., 1983; Pastelín et al., 2017). Similarly, the prostate is also innervated by parasympathetic pelvic nerves, sympathetic nerves T12-L3 and sensory nerves DRG L5-L6 (Ahuja, 2011; McVary et al., 1998; White et al., 2013). Innervation of the pancreas consists of parasympathetic vagus nerves, sympathetic nerves from celiac and superior mesenteric ganglia, and sensory nerves from NG and DRG T9-T13 (Woods and Porte, 1974). Lungs are innervated by parasympathetic vagus nerves, sympathetic nerves originating from T1-T6, and sensory nerves originating from NG and DRG T1-T6 (Belvisi, 2002; Springall et al., 1987).

The head and neck, breast and skin are instead predominantly innervated by sensory nerves. The throat, mouth and nose, which are common sites of squamous cell carcinoma origin, are innervated mainly by sensory nerves from the trigeminal ganglia at the base of CN V and the DRG of C2-C3 (Waxenbaum et al., 2021). Nerve supply to the head and neck also includes parasympathetic nerves from CN VII and CN IX and sympathetic nerves from the super-cervical ganglia of the sympathetic chain (Vilensky et al., 2015). The breast is innervated primarily by sensory nerves from C3-C4 and T3-T6, and secondarily by sympathetic nerves from T1-T5, which cover the ducts (Liu and Krassioukov, 2013; Sarhadi et al., 1996). Similarly, sensory neurons from DRG innervate all layers of the skin, whereas sympathetic neurons from the sympathetic chain only innervate the dermis and glands (Roosterman et al., 2006). Notably, nerve supply to the skin is not limited to certain ganglia but originates from ganglia all over the body.

Organ nerve supply in mouse is relatively similar to that in human. The mouse spinal cord consists of 34 segments: eight cervical (C1-C8), 13 thoracic (T1-T13), six lumbar (L1-L6), four sacral (S1-S4) and three coccygeal (Co1-Co3) (Sengul et al., 2012). The mouse GI tract is innervated by sensory NG and DRG L3-S3, by sympathetic celiac, superior mesenteric and inferior mesenteric ganglia, and by parasympathetic vagus and pelvic nerves (Phillips and Powley, 2007). Sympathetic celiac and superior mesenteric ganglia, and parasympathetic pelvic nerves supply the ovaries (Pastelín et al., 2017). The prostate is innervated by sensory DRG T10-S1, sympathetic chain T11-S1 and parasympathetic pelvic nerves (Garrett et al., 2021). Pancreas innervation in mouse is identical to that in human, consisting of sensory nerves from NG and DRG T9-T13, sympathetic nerves from celiac and superior mesenteric ganglia, and parasympathetic nerves from vagus nerves (Makhmutova and Caicedo, 2021). The murine lung is innervated by thoracic sensory DRG, sympathetic chain C4-T3 and parasympathetic vagus nerves (Garrett et al., 2021), while the head and neck are innervated by sensory trigeminal ganglia, sympathetic superior cervical ganglia and parasympathetic cranial nerves (Scott-Solomon et al., 2021; Vermeiren et al., 2020). Unlike in humans, murine mammary glands consist of ten glands that are spread across the cervical, thoracic, abdominal and inguinal sections of the body and thus receive nerve supply from sensory NG as well as corresponding DRG and sympathetic chain ganglia (McCallum et al., 2020; Ventrella et al., 2021). Similarly, the skin receives innervation from sensory DRG and sympathetic chain ganglia in the surrounding area (Boada and Woodbury, 2007; Botchkarev et al., 1998). When starting to study the role of nerves in a cancer type in mouse models, it is important to be aware that each organ receives input from different types of nerves originating from different regions in the spinal cord.

Although innervation of healthy tissues is well characterized, how the neuronal population changes during tumorigenesis remains poorly understood. Cancer cells themselves have been shown to upregulate pan-neuronal markers that are traditionally used to assess neuronal lineages. For example, β3-tubulin and PGP9.5 (also known as UCHL1) are expressed in breast, melanoma and prostate cancer cells (Goto et al., 2015; Kanojia et al., 2015), and mature neuron markers, including MAP2 and MAPT, are expressed in breast, gastric and lung cancer cells (Caillet-Boudin et al., 2015; Yang et al., 2019a). Thus, we cannot rely on mining publicly available gene expression datasets of bulk tumor tissues to determine the presence and abundance of neuronal populations in human tumors. Instead, characterization of the neuronal subtypes present in tumors needs to be done by immunostaining for at least a pan-neuronal marker and a subtype-specific marker, for example, TH for sympathetic, CHAT for parasympathetic and TRPV1 for sensory neurons. In addition, the gold standard for determining the origin of innervation is retrograde tracing with tracer molecules or viral transfection (Saleeba et al., 2019). A recent paper used neural tracing to show that the increased sensory innervation in high-grade serous ovarian carcinoma stems not only from the local thoracic and lumbar spinal nerves, but also from NG of the cranial nerves (Barr et al., 2021). Unfortunately, although retrograde tracing has been extensively used in neuroscience, it has not yet been widely adapted in the context of cancer.

Reavis et al. (2020) concisely summarized the current literature on the types of nerves that have been studied in various cancers. In most cases, the neuronal population in a tumor is similar to that originally present in the healthy tissue. For example, gastric tumors are densely innervated by autonomic nerves (Zhao et al., 2014), while head and neck tumors show high levels of sensory nerve innervation (Madeo et al., 2018). However, several studies have also shown that neuronal makeup can change when a tumor arises within a tissue, as discussed in more detail below. In summary, it is important to be aware of the neuronal populations present in tumor types to motivate the rational design of relevant experiments to study nerve–cancer crosstalk.

## Mechanisms of increased innervation in cancer

In several organs, the presence of a tumor has been associated with increased innervation (Albo et al., 2011; Allen et al., 2018; Ayala et al., 2008; Huang et al., 2014; Madeo et al., 2018; Partecke et al., 2016; Shao et al., 2016; Zhao et al., 2014). There are several known and hypothesized mechanisms by which tumorigenesis leads to an increase in nerve density. First, cancer cells can release axon guidance molecules and growth factors that induce the outgrowth of existing nerves. For example, Madeo et al. (2018) showed that patient-derived head and neck squamous cell carcinomas pack ephrin B1 within exosomes, which is then released to stimulate axonogenesis of cultured neuronal-like PC12 cells. Similarly, breast and prostate cancer cells secrete NGF to induce neural outgrowth of PC12 cells and of the sensory neuron cell line 50B11 *in vitro* (Pundavela et al., 2014; 2015). The axon guidance molecule semaphorin 3D is secreted by pancreatic cancer cells to interact with neuronal plexin D1, directing the *in vivo* innervation of the pancreas in a orthotopic pancreatic ductal adenocarcinoma mouse model (Jurcak et al., 2019). Disrupting the interaction between semaphorin 3D and its receptor plexin D1 reduced the invasion of cancer cells towards DRG sensory neurons *in vitro* and the extent of metastasis *in vivo*. Furthermore, neurogenesis is closely tied to angiogenesis – a hallmark of cancer – as both share common pathways and can regulate one another (Boilly et al., 2017); VEGF-A is released by breast cancer cells and induces outgrowth and axonal branching of 50B11 cells *in vitro* (Austin et al., 2017). Although these studies suggest that the presence of a tumor can induce axon growth of existing nerves, it is not well known whether tumors can induce neurogenesis or drive the generation of new neurons.

Second, the fact that some tumor tissues contain nerve types that are not readily present in healthy tissues suggests that these nerves might have a different origin. For example, parasympathetic nerve fibers were detected in breast tumors from patients and in breast cancer xenografts, but are absent from healthy breast (Kamiya et al., 2019). In a recent preprint, Kovacs et al. (2020) found that sensory fibers were abundant in ovarian tumor tissues of a mouse model of ovarian cancer but absent from normal ovaries. Neural progenitor and immature neuron markers such as nestin and doublecortin are highly expressed in the tumor mass, suggesting that neuronal maturation may be occurring within tumors (Ayanlaja et al., 2017; Ishiwata et al., 2011). A recent study in the Hi-Myc transgenic mouse model of prostate cancer proposes an explanation: early in cancer development, doublecortin^+^ neural progenitors from the brain subventricular zone cross the blood–brain barrier and infiltrate prostate tumors (Mauffrey et al., 2019). These neural progenitors then differentiate into sympathetic neurons that innervate the prostate tumor. More importantly, Mauffrey et al. (2019) also found doublecortin^+^ cells in breast tumors and metastasis sites such as colon, liver and lung. The final fate of these cells, however, remains unclear. Therefore, more work is needed to better understand how nerves that are not originally present within a tissue find their way when a tumor arises in that same tissue.

Third, transdifferentiation of tumor or neural cells could be a potential source of new nerves. Gene expression profiles of highly aggressive cancer subtypes of the breast (Jézéquel et al., 2019), prostate (Zhang et al., 2016) and ovary (Yang et al., 2019b) exhibit signatures of stemness and neural development pathways. Indeed, a fraction of cancer stem cells isolated from human colorectal and gastric adenocarcinomas can be induced to differentiate into parasympathetic and sympathetic neurons *in vitro*, and innervate and support tumor progression when injected *in vivo* (Lu et al., 2017). Under serum-deprived conditions, LNCaP prostate cancer cells undergo neuronal transdifferentiation, demonstrated by the loss of prostate cancer cell markers, such as androgen receptor and prostate-specific antigen, and by the gain of neuronal traits, such as neurite extension and expression of neuronal gene signatures (Farach et al., 2016). Further, new neuronal subtypes can originate from the transdifferentiation of existing nerves in the tumor mass. Injecting sensory nerves within head and neck tumors with extracellular vesicles from p53 (also known as TP53)-deficient cancer cells *in vivo* reprogrammed these nerves into norepinephrine-producing adrenergic nerves (Amit et al., 2020). Owing to the various ways in which tumors can induce innervation, the intratumoral neuronal population likely comes from a mix of different origins that may depend on the cancer type.

## Peripheral nerves have tumor-specific effects on cancer progression

The effects that nerves have on cancer progression depend on the type of tumor as well as the neural subtypes present. Until now, most of the research in the field has focused on how the autonomous nervous system affects cancer through the release of soluble cues: norepinephrine and acetylcholine. Sympathetic nerves support tumor growth and metastasis in prostate (Magnon et al., 2013), breast (Sloan et al., 2010) and pancreatic cancer (Allen et al., 2018). Sympathetic nerves release norepinephrine, which signals through β-adrenergic receptors on cancer or stromal cells in the TME and activates downstream pro-tumor pathways. In prostate cancer, sympathetic nerve-mediated β-adrenergic signaling aids tumor initiation by promoting cancer cell survival (Magnon et al., 2013). β-adrenergic signaling also facilitates angiogenesis through a metabolic switch to activate glycolysis in prostate cancer endothelial cells (Zahalka et al., 2017). In pancreatic cancer, nerve-driven β-adrenergic signaling creates a feedforward loop in which cancer cells produce more NGF and BDNF, which in turn drives innervation and increases the release of norepinephrine (Allen et al., 2018; Renz et al., 2018a).

Sympathetic nerves also regulate tumor growth via the immune system. In a breast cancer mouse model, chronic stress or sympathetic stimulation increased inflammation, as measured by M2 macrophage infiltration, and immune invasion through PD-L1 (also known as CD274) expression (Kamiya et al., 2019; Sloan et al., 2010). The role of β-adrenergic receptors in lung and ovarian cancer has also been extensively studied (Huang et al., 2018; Nilsson et al., 2020; Thaker et al., 2006). Activation of adrenergic receptors on cancer cells increases the intracellular cyclic AMP concentration, which in turn activates pro-tumor signaling pathways such as the PKA (also known as PRKA) and MAPK pathways. However, whether sympathetic nerves contribute to the activation of adrenergic receptors is not well understood. It should be noted that norepinephrine is also secreted by the adrenal medulla as a hormonal response to stress, which can complicate differentiating between sympathetic- and adrenal-driven contributions to adrenergic signaling. So far, studies in multiple cancer types suggest that sympathetic nerves are pro-tumorigenic and that targeting the β-adrenergic receptor is a promising strategy to inhibit tumor progression in multiple cancer types. Previous clinical trials in melanoma (Gandhi et al., 2021) and breast cancer (Hiller et al., 2020) that evaluated the use of the β-blocker propranolol in combination with surgery or chemotherapy have shown an increase in IFNγ and immune infiltration. In addition, there are currently several ongoing clinical trials studying the effect of propranolol in various cancers (NCT03384836, NCT03152786, NCT04848519, NCT04682158). The role of sympathetic nerves in tumor progression is therefore well established and is the first to lead to a clinical trial targeting nerve-driven effects on tumor cells.

The effect of parasympathetic nerves on tumor progression is also driven by the release of their main neurotransmitter, acetylcholine. Unlike sympathetic nerves, parasympathetic nerves have opposing effects in different tumor types: parasympathetic innervation is pro-tumorigenic in prostate and gastric cancer (Magnon et al., 2013; Zhao et al., 2014) but has anti-tumor effects in breast and pancreatic cancer (Kamiya et al., 2019; Renz et al., 2018b). In gastric cancer, acetylcholine activates muscarinic receptors on cancer cells and their downstream Wnt signaling, promoting stemness and tumorigenesis (Zhao et al., 2014). Here, a feedforward loop also exists in which acetylcholine induces NGF secretion by gastric epithelial cells, which drives more parasympathetic innervation (Hayakawa et al., 2017). In prostate cancer, acetylcholine activates muscarinic receptors on stromal cells to disrupt the basement membrane and encourage metastasis (Magnon et al., 2013). In contrast, acetylcholine-mediated activation of muscarinic receptors on pancreatic cancer cells inhibits tumor progression by downregulating MAPK/EGFR and PI3K/AKT pathways (Renz et al., 2018b). In breast cancer, acetylcholine activates muscarinic receptors on tumor-infiltrating lymphocytes to reduce their expression of PD-1 (also known as PDCD1) *in vivo*, leading to suppressed tumor growth due to the removal of the immune checkpoint and consequent increase in the anti-tumor immune response (Kamiya et al., 2019). Although several studies have shown that parasympathetic nerves regulate various aspects of tumor progression via cancer cells themselves and via the TME, these effects appear to be cancer-type specific and warrant further investigation.

Sensory nerves affect tumor progression in pancreatic, breast, skin and prostate cancer (Ayala et al., 2001; Keskinov et al., 2016; Lei et al., 2016; Saloman et al., 2016). Removing sensory nerves from the pancreas via neonatal capsaicin injection delayed cancer onset and progression in an autochthonous mouse model expressing mutant KRAS (Saloman et al., 2016). Co-injection of murine DRG sensory neurons with B16 melanoma cells accelerated tumor growth in a xenograft model (Keskinov et al., 2016). In *in vitro* models, co-culture with DRG sensory neurons enhances the proliferation and survival of pancreatic cancer cells compared to cancer cell monoculture (Dai et al., 2007). Our laboratory's work has recently shown that sensory nerves can drive migration and metastasis in triple-negative breast cancer and induce significant changes in the gene expression of cancer cells. These cancer cells migrate directly along nerves, an interaction driven by expression of the axon guidance receptor plexin B3 expressed by tumor cells (Le et al., 2022). Moreover, the sensory nervous system is a known immune regulator (Pinho-Ribeiro et al., 2017). In melanoma and breast cancer, activation of sensory nerves by a TRPV1 agonist or via chemogenetic modulation increases the recruitment of cytotoxic T cells and IL-17 production in the primary tumor (Costa et al., 2021; Erin et al., 2022). These results suggest that, although the presence of sensory nerves is generally pro-tumorigenic, activating these nerves by stimulating their electrical properties is a potential treatment strategy that can manipulate the immune landscape to be anti-tumorigenic.

Another way the PNS can support metastasis *in vivo* is through perineural invasion, a process in which cancer cells invade and migrate along the nerve sheath (Liebig et al., 2009). *In situ*, nerves are often organized into bundles consisting of glial cells, oligodendrocytes, endothelial cells and the extracellular matrix to facilitate neuronal functions. Tumor cells of head and neck, prostate, gastric and pancreatic cancer utilize this structure as a route of metastasis (Chen et al., 2019; Marchesi et al., 2010). As described earlier, nerves that infiltrate tumors appear as bundles and individual nerve twigs, but not nerve sheaths (Austin et al., 2017; Madeo et al., 2018; Pundavela et al., 2015; Reavis et al., 2020). *In vitro* models co-culturing DRG sensory nerve and breast and prostate cancer cells have shown that individual nerve fibers can provide physical support for the migrating cells (Ayala et al., 2001; Lei et al., 2016). It is still unclear whether this phenomenon is similar to perineural invasion, or if it occurs *in vivo*.

Lastly, neurons can form synaptic connections with cancer cells. In gliomas, neuron-to-glioma synapses are modulated by the glutamate receptor α-amino-3-hydroxy-5-methyl-4-isoxazolepropionic acid (AMPA) receptor. Synaptic input through this receptor leads to depolarization of glioma cells by an influx of intracellular calcium, which activates downstream pathways to then induce cancer cell proliferation and invasion (Venkataramani et al., 2021). Synaptic communication can also occur with cancer cells that do not originate from the central nervous system (CNS). The glutamate-gated cation channel N-methyl-D-aspartate (NMDA) receptor is overexpressed in breast cancer cells, and even to a higher degree when these cells invade the brain (Zeng et al., 2019), which can be activated by glutamate that leaks from a neighboring synaptic cleft. The connection, termed pseudo-synapse, enables breast cancer cells to leverage glutaminergic signaling to support the metastatic colonization in the brain. In an allograft mouse model of triple-negative breast cancer, the chronic electrical activity of primary breast tumors was also significantly higher, displaying tenfold more neural spikes than normal breast tissues (McCallum et al., 2020). In the same study, stimulation of the vagus nerve led to action potentials within the tumor, suggesting a neural connection between the vagus nerve and breast tumor. However, this study did not provide conclusive evidence of synaptic communication between peripheral nerves and cancer cells.

Overall, there is an increasing amount of literature demonstrating how different types of nerves can contribute to tumor progression, both by directly interacting with and regulating the properties of cancer cells via soluble cues, electrical cues or cell–cell contact, and by influencing the function and composition of the local TME.

## Current approaches to study nerve–cancer interactions

Our knowledge of the mechanisms by which nerves contribute to cancer progression depends on the experimental models, both *in vivo* and *in vitro*. Given the complexity of the neural compartment, it is critical that experimental approaches faithfully recapitulate aspects of the nerve–cancer interaction that are of interest. In this section, we summarize the current models to study innervation in cancer, identify their advantages and disadvantages, and highlight the technologies or methodologies that could bridge those gaps.

### *In vivo* models

**Fig. 2.**
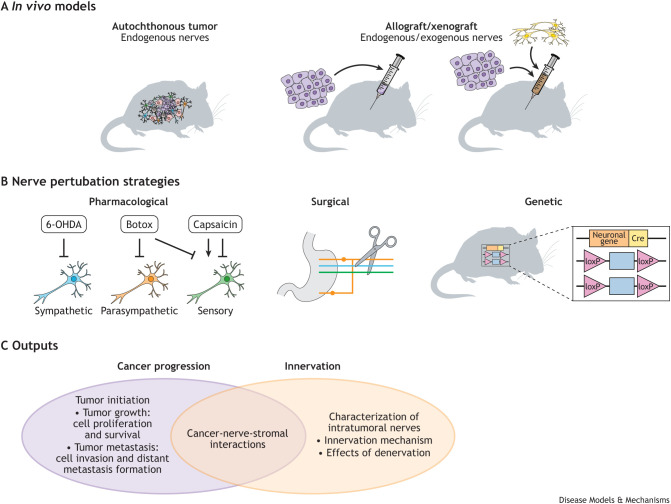
***In vivo* models to study nerve–cancer interaction.** (A) Autochthonous and allograft/xenograft mouse models are used to study nerve–cancer interaction. (B) *In vivo* nerve perturbation strategies include pharmacology, denervation surgery and genetic engineering. (C) Outputs of *in vivo* models include studying how nerves affect cancer progression, how innervation happens in cancer, and the effects nerves have on stromal cells. 6-OHDA, 6-hydroxy-dopamine.

*In vivo* mouse models are important in cancer research, because they can recapitulate the tumor growth and metastasis processes in a physiological environment, with or without an intact immune system. When using mouse models to study nerve–cancer interactions, researchers need to consider the type of mouse, nerve perturbation approaches and experimental outputs (Fig. 2). Autochthonous models in which tumors develop within the murine tissues spontaneously or after induction provide the most accurate model for understanding the development and role of tumor innervation. These can determine the identity of nerves endogenously present in a tumor, and track innervation density and progression as the tumors grow. They can also be used to study the effect of endogenous nerve depletion on tumor progression. For example, in Magnon et al. (2013), which we discussed above, the authors performed autonomic denervation at different time points in an autochthonous Hi-Myc prostate cancer mouse model and concluded that sympathetic nerves support tumor initiation, whereas parasympathetic nerves support invasion. However, not all tumor types have genetic mouse models readily available. These models are also costly and significantly harder to modify, requiring a new mouse strain for each new target gene of interest.

As nerve supply to each organ is unique, orthotopic allograft and xenograft models are also attractive to study cancer–nerve interactions. First, they allow for the use of both mouse and human cell lines, which can be easily genetically modified in culture before implantation. Using human tumor cells in a mouse host also allows researchers to take advantage of a species difference between the human graft and the endogenous murine nerves for mechanistic studies using antibodies, species-specific RNA sequencing (RNAseq) or proteomics. Second, neurons can be co-injected with tumor cells to manipulate their amount and identity. In our recent study, also discussed above, we co-injected the triple-negative human breast cancer cells MDA-MB-231 and murine DRG sensory neurons into immunodeficient mice and found an increase in innervation of the primary tumor and increased lung metastasis (Le et al., 2022). However, given that nerves influence tumor initiation, xenograft models might not fully replicate the timing and context of the nerve–cancer interaction. These models also require immunodeficient mice to support the growth of human cancer cells and neurons, which may interfere with the known role of nerves in regulating the immune response (Costa et al., 2021; Kamiya et al., 2019). Finally, combinations of xenograft and genetically engineered mice can dissect the relationship between nerves, stromal and cancer cells. For example, to study whether parasympathetic nerves interact with stromal or cancer cells directly, PC-3 prostate cancer cells with an intact muscarinic receptor were implanted into mice engineered to lack the muscarinic receptor (Magnon et al., 2013). Addition of the muscarinic receptor agonist carbachol significantly increased lymph node metastasis in muscarinic receptor-sufficient mice, while having no effect on deficient mice, suggesting that acetylcholine-mediated metastasis acts through stromal cells instead of cancer cells. Although these *in vivo* approaches are relatively straightforward to implement and have distinct advantages in terms of dissecting tumor versus host effects, they require in-depth characterization of the nerves *in vivo* in terms of their survival and function.

In mouse models, nerves can be perturbed using three main methods: pharmacological, surgical and genetic (Fig. 2B). Pharmacological approaches can either activate or deactivate nerves or induce nerve death. 6-Hydroxy-dopamine (6-OHDA) and 1-methyl-4-phenyl-1,2,3,6-tetrahydropyridine are neurotoxins that specifically target sympathetic noradrenergic nerves (Schober, 2004). 6-OHDA is non-toxic to cancer cells and therefore has been used extensively to inactivate sympathetic signaling in tumor innervation studies (Magnon et al., 2013; Szpunar et al., 2016). Botulinum toxin type A (Botox) is another effective general neurotoxin that blocks neurotransmitter release in parasympathetic and sensory nerves (Dressler and Saberi, 2005). Sensory nerves can be targeted via TRPV1 with capsaicin and its analogs resiniferatoxin and olvanil (Erin et al., 2022). However, depending on dosage, capsaicin can either induce, desensitize or completely ablate sensory nerves (Fischer et al., 2020; Szolcsányi, 2014). Thus, although the effects of these drugs on nerve function have been well established, when using them to explore cancer–nerve interactions, it is important to use appropriate controls and to monitor for direct effects of these toxins on cancer cells.

A surgical approach can locally ablate nerves in an organ of interest. In the context of cancer, this method has been used in the gut, prostate, breast and skin (Kappos et al., 2018; Magnon et al., 2013; Ostrowski et al., 2011; Zhao et al., 2014). Although this procedure is highly effective at removing nerves, it is often invasive and requires specific training. Nerve fibers are also not distinct, making it difficult to separate individual nerves; nerve supply to organs often comes from the plexuses, which are gathering points for nerves of multiple types. For example, the hypogastric nerve innervating the bladder and prostate is thought to be sympathetic, yet it also contains sensory nerves (Lorenz et al., 2011). Therefore, it is important to assess the amount and identity of the remaining nerves after the denervation procedure. Further, surgery on its own, especially close to the organ of interest, can cause local inflammation with its own distinct effects on cancer progression. Therefore, these types of experiments require surgical sham controls to ensure the validity of the results.

Finally, denervation can be achieved through genetic manipulation to alter nerve activity or survival. TRPV1 or TH knockout mice are commercially available and have been used in neuroscience studies (Garami et al., 2011; Hnasko et al., 2007). It should be noted that these mice retain peripheral sensory and sympathetic nerves – only their activity is inhibited. One limitation of these mouse models is that the transgenes are usually engineered in a certain genetic background. To allow research in the context of cancer, these mice need to be crossed with the genetic cancer model strain of the same background, or, for allograft and xenograft experiments, require the use of tumor cells that would implant in such background. Bypassing this limitation, one study used adeno-associated virus to manipulate sympathetic nerve activity in a breast cancer model (Kamiya et al., 2019). In this study, genes encoding a fluorophore to label neurons, diphtheria toxin to ablate nerves, and sodium channels and a fluorescent calcium indicator to modulate neuron firing were transfected to sympathetic nerves with high efficacy by placing them under the control of the TH promoter. As a result, this group was able to specifically target TH^+^ sympathetic nerves for ablation or stimulation. There are also more sophisticated knockout mouse lines, in which researchers use the Cre-loxP system and designer receptors exclusively activated by designer drugs (DREADD) to chemogenetically target specific neural populations. In a melanoma model, Nav1.8-Cre mice, which express Cre only in Nav1.8^+^ (also known as SCN10A^+^) sensory neurons, were crossed with mice expressing mutant G protein-coupled receptors that can either induce or inhibit sensory neuron activity. Administering clozapine-N-oxide, a specific ligand of the mutant G protein-coupled receptors, to the resulting mouse activated said receptors and subsequently silenced or activated sensory nerves (Costa et al., 2021). These genetic approaches provide many advantages in terms of the spatial and temporal control of gene expression, allowing researchers to specifically regulate neural activity at precise stages of tumor progression.

Overall, these nerve perturbation methods (surgical, chemical, genetic) are more focused on decreasing or removing nerve activity. All methods involving cell death unavoidably alter the microenvironment, as an inflamed TME is a potent pro-tumor characteristic (Greten and Grivennikov, 2019). Further, nerve damage can trigger endogenous nerve regeneration, which could have unintended side effects on tumors (Boilly et al., 2017; Geuna et al., 2016). Recent studies have demonstrated that it is possible to inject nerves into murine tumors. Studies show that injecting DRG sensory neurons can successfully increase the nerve density within a tumor and leads to increased metastasis in breast cancer and melanoma, respectively (Keskinov et al., 2016; Le et al., 2022). However, confirming the identity and functionality of these newly introduced nerves can be challenging. They are often dissected from the same mouse strain to reduce host immune response, thus making staining for strain-specific antibodies ineffective. Primary neurons need to be marked before injection, either by transfection to express fluorescent proteins, which can have issues with transfection efficiency and toxicity, or isolated from transgenic mice with fluorescently tagged neurons. Therefore, more methods are needed to precisely tune the abundance and activity of individual nerve populations *in vivo* without disrupting the tumor population.

### *In vitro* models

**Fig. 3.**
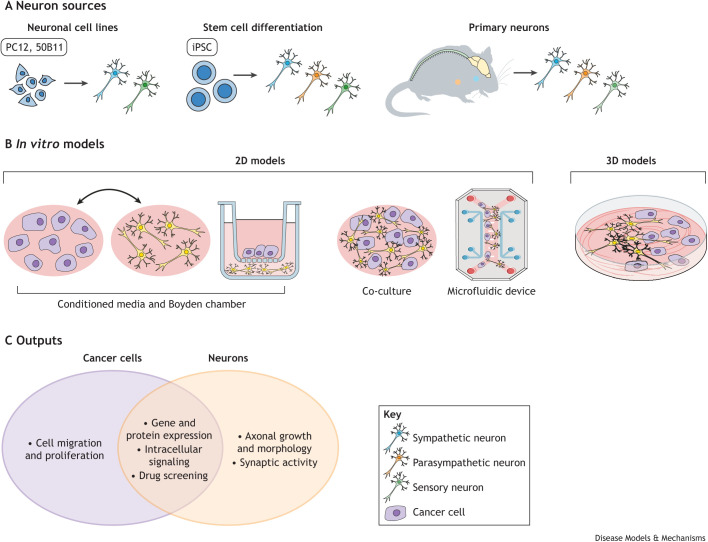
***In vitro* models to study nerve–cancer interaction.** (A) Neuron sources: immortalized cell lines, induced pluripotent stem cell (iPSC) differentiation and primary neurons from rodent dissection. (B) Current *in vitro* models of nerve–cancer interaction include two-dimensional (2D) cultures and exchanging conditioned media between the two cell types, which can be achieved either via media transfer between separate culture vessels or in Boyden chamber-based assays. Alternatively, direct co-culture of the two cell types is possible within the same vessel and in microfluidic devices. In three-dimensional (3D) co-culture models, the cells are individually suspended in Matrigel, an extracellular matrix mimetic. (C) *In vitro* models allow thorough investigation into the mechanism of nerve–cancer crosstalk by studying the individual effects of cancer cells and neurons, as well as their gene expression and signaling pathways in response to reciprocal stimuli, multi-omics and electrical communication between the two cell types.

Unlike *in vivo* models, considerations for *in vitro* models mainly revolve around what types of cells and assays are appropriate for the research questions (Fig. 3). Both mouse and human cancer cell lines or primary cultures of human cancer cells can, to some extent, recapitulate the heterogeneous nature of cancer. Sourcing neuronal cells for *in vitro* studies, however, is more complicated. As neurons are terminally differentiated, they no longer proliferate, which makes them challenging to maintain in culture. The most common neuronal cell line used in cancer research is PC12, derived from a neuroendocrine tumor in rat adrenal medulla (Kaduri et al., 2021; Kovacs et al., 2020 preprint; Madeo et al., 2018; Pundavela et al., 2015). PC12 cells can be terminally differentiated into sympathetic-like neurons in the presence of NGF. These cells are well established and easy to culture, and they have primarily been used to model CNS dopaminergic neurons in Parkinson's disease. However, PC12 cells are unable to develop synaptic endings and have highly variable morphology that depends on passage number (Das et al., 2004; Wiatrak et al., 2020). Thus, PC12 cells are only suitable to study the effects of secreted factors on the interactions between cancer cells and sympathetic nerves. For studying the effects of sensory neurons on cancer cells, the 50B11S cell line is derived from rat DRG neurons and can be induced to differentiate into functional sensory neurons (Bhattacherjee et al., 2014). However, they die 72 h after differentiation, which limits the types of experiments and functional outcomes that can be measured (Geuna et al., 2016; Pittier et al., 2005). Another option is deriving peripheral nerves from induced pluripotent stem cells (iPSCs). Researchers can efficiently derive sympathetic, parasympathetic and sensory neurons from iPSCs following well-established protocols (Kirino et al., 2018; Takayama et al., 2020; Guimarães et al., 2018). However, this approach can be time consuming, and, to our knowledge, no study has leveraged iPSC-derived neurons in the context of cancer. Primary neurons from rodents can also be maintained in culture. Peripheral ganglia can be dissected and cultured, providing fully functional neurons. The most common primary neurons used in the context of cancer are sensory nerves from DRG, owing to their ease of dissection and culture. However, DRG contain both somatosensory and visceral neurons, which innervate bones or muscles and internal organs or blood vessels, respectively. When using these unsorted neurons, investigators need to be careful when drawing conclusions without further validation. Sympathetic nerves can be dissected from the superior cervical ganglia in the neck and the stellate ganglia in the rib (Johnson, 2001; Scherschel et al., 2020; Zareen and Greene, 2009). Parasympathetic nerves can be dissected from intracardiac ganglia in the heart (Hoard et al., 2007). A key disadvantage of primary neurons is that they survive only up to a few weeks in culture and cannot be frozen, requiring fresh dissections to supply new cells. Therefore, there are multiple sources of cells that can be used to improve upon our current *in vitro* models of nerve–cancer interaction, each with its distinct advantages and shortcomings.

Most cancer cell–nerve interaction studies have focused on evaluating the role of soluble factors through which these cell types communicate. As a result, culture models in these studies have been limited. They either determined the effects of conditioned media from one cell type to another (Austin et al., 2017; Keskinov et al., 2016; Madeo et al., 2018) or cultured cancer cells and neurons in a Boyden chamber, in which cancer cells are separated from nerves by a porous membrane (Jurcak et al., 2019; Pundavela et al., 2015). Although these models have been very useful in evaluating the effects of secreted factors from neurons on cancer cells and vice versa, they do not allow the study of direct cell–cell contact between the two cell types. In monolayer co-culture models, in which the cells are in direct contact, cancer cells can utilize nerve fibers as physical structures on which to move (Ayala et al., 2001; Lei et al., 2016), further prompting investigation into diverse modes of cell–cell communication and their biological consequences. Our own work has recently shown that tumor cells can directly interact with sensory nerves *in vitro*, and use them to migrate at significantly faster speeds (Le et al., 2022). A recent study demonstrated that breast cancer cells that have metastasized to the brain formed a pseudo-synapse with cortical neurons, suggesting that proximity plays an important role in nerve–cancer crosstalk (Zeng et al., 2019). These studies suggest that more direct co-culture systems are needed to more broadly determine the role of tumor cell–nerve interactions. Unlike monolayers, three-dimensional (3D) co-culture models better mimic the tumor microenvironment. Two groups used such models to investigate pancreatic cancer–nerve interactions. The authors prepared separate suspensions of MIA PaCa pancreatic cancer cells and DRG sensory neurons in Matrigel, which separated the two cell types and allowed the researchers to observe their interactions in an extracellular matrix-like structure. In both studies, pancreatic cancer cells exhibited directed migration towards the sensory nerve fibers (Bapat et al., 2016; Dai et al., 2007). Given the importance of studying tumor cell behaviors in 3D models *in vitro*, it is clear that more studies incorporating 3D approaches and a functional extracellular matrix component are necessary and will be valuable in further dissecting tumor–nerve interactions.

Most studies published to date have focused on short time points (7-14 days). However, advances in disease modeling have enabled researchers to develop model systems that could sustain CNS neuron function for extended periods of time, from 4 months to 2 years (Koroleva et al., 2021; Rouleau et al., 2020). If and when applied to the study of cancer innervation, these models will allow researchers to study how PNS nerves influence the tumor progression process *in vitro* at timescales that better recapitulate the human disease. An additional challenge of current nerve–cancer co-culture models is that their output is often limited to proliferation and migration. Gene expression analysis through microarray has provided a glimpse of the mechanism of nerve–cancer interactions (Dai et al., 2007; Zhao et al., 2014). However, microarrays have long been surpassed by more comprehensive -omics approaches, such as RNAseq and proteomics, but these have not been widely applied to investigate nerve–cancer crosstalk. Our group used species-specific RNAseq, which showed that triple-negative breast cancer cells upregulate immune-, extracellular matrix- and migration-related pathways when co-cultured with DRG sensory neurons (Le et al., 2022). This work demonstrates that co-culture *in vitro* models is amenable to -omics approaches. Furthermore, these *in vitro* models allow researchers to measure and modulate the electrical properties of neurons in co-culture, or of cancer cells themselves, with classical methods such as patch clamp, fluorescent calcium indicators and ion manipulation (Jin et al., 2012; Mansor and Ahmad, 2015). Recently developed methods now allow the fabrication of electrodes at nanoscale. These could be embedded in the surface of cell culture vessels or microfluidic chambers, enabling seamless electrical monitoring and stimulation (Dvir et al., 2011; Ju et al., 2020). Such settings provide enhanced spatial and temporal control, as electrical stimulation and measurement can be performed locally at any point, with predetermined magnitude, frequency and pattern. Applying these approaches to current models could lead to a deeper understanding of nerve–cancer interactions.

## Conclusions

How peripheral nerves affect cancer is a complex, yet understudied, area of research. Work published so far demonstrates that nerves interact with tumor cells either directly by releasing growth factors and neurotransmitters, providing physical support and electrical activity, or indirectly via effects on angiogenesis or the immune system. However, the detailed mechanisms of nerve–cancer interactions are still not completely known, and understanding them will require applying available technological advances to study neuronal function that were developed by neuroscientists and optimizing novel approaches to couple existing methods with current cancer models. Further, dissecting how nerves interact with and regulate the myriad of cells and components in the TME will also be critical. Studies have shown that nerves can affect angiogenesis and recruitment of certain immune cells, but few studies have investigated how nerves impact other cell types, such as fibroblasts or adipocytes, or the composition of the extracellular matrix, all of which can contribute to cancer progression.

Nerve presence is correlated with aggressive disease and poor prognosis; therefore, early detection of tumor innervation could be a sign that further intervention and monitoring are needed. There are already established methods to non-invasively detect neuronal presence. Indeed, in the brain, N-acetyl aspartate is considered to be of neuronal origin, and high levels detected by magnetic resonance spectroscopy signify nerve presence and function (Mabray et al., 2015). Thus, detecting N-acetyl aspartate could be adapted for solid tumors to characterize nerve density and therefore disease progression. In addition, nanoparticles with nerve-binding peptide NP41 can also be used as a contrast agent in magnetic resonance imaging, and this approach has been used to visualize innervation in prostate cancer in mice (You et al., 2020). Gaining a clearer understanding of how nerve density and activity affect prognosis and progression is critical to the implementation of these methods in the clinic.

Lastly, nerves are also an attractive target for novel treatments for cancer patients. As shown in mouse models, denervation can reduce tumor mass and cancer progression (Magnon et al., 2013; Zhao et al., 2014). Thus, non-resectable tumors could be denervated to control their growth and reduce metastasis (Demir et al., 2020). In addition, existing neurological drugs can be repurposed to target intratumoral nerves and used alongside traditional chemotherapies. Aside from β-blockers, there is evidence that antidepressant drugs such as selective serotonin reuptake inhibitors, norepinephrine–dopamine reuptake inhibitors and tricyclic antidepressants have anti-tumor effects and prolong patient survival (Li et al., 2022; Zingone et al., 2017). Ultimately, a better understanding of the mechanisms of tumor innervation are needed to establish nerve presence as a hallmark of cancer and a viable therapeutic target.
